# TGF-β1/FGF-2 signaling mediates the 15-HETE-induced differentiation of adventitial fibroblasts into myofibroblasts

**DOI:** 10.1186/s12944-015-0174-3

**Published:** 2016-01-05

**Authors:** Li Zhang, Yan Chen, Guixia Li, Minggang Chen, Wei Huang, Yanrui Liu, Yumei Li

**Affiliations:** Department of Pharmacology, Harbin Medical University-Daqing, Xinyang Road 39, Daqing, Heilongjiang Province 163319 China; Daqing Qil Fields General Hospital, Heilongjiang, Daqing, Heilongjiang Province 163319 China; Biopharmaceutical Institute of the Heilongjiang Academy of Medical Sciences, Harbin, Heilongjiang Province 150081 China

**Keywords:** Pulmonary adventitial fibroblasts, 15-HETE, FGF-2, Myofibroblasts, p38/Egr-1

## Abstract

**Background:**

Pulmonary adventitial fibroblasts (PAFs) are activated under stress stimuli leading to their differentiation into myofibroblasts, which is involved in vessel remodeling. 15-HETE is known as an important factor in vessel remodeling under hypoxia; however, the role of 15-HETE in PAF phenotypic alteration is not clear.

**Results:**

The effect of 15-HETE on PAF phenotypic alterations was investigated in the present study. PAFs were treated with 15-HETE (0.5 μM) for 24 h, and the myofibroblast marker α-smooth muscle actin (α-SMA) was analyzed. The 15-HETE induced α-SMA expression and cell morphology. 15-HETE upregulated FGF-2 levels in PAFs, and knockdown FGF-2 by siRNAs blocked the enhanced α-SMA expression induced by 15-HETE. p38 kinase was activated, and blocked depressed 15-HETE-induced FGF-2 expression. The downstream of p38 pathway, Egr-1 activation, was also raised by 15-HETE treatment, and silenced Egr-1 suppressed the 15-HETE-induced upregulation of FGF-2. TGF-β1 was upregulated with FGF-2 treatment, and α-SMA expression induced by FGF-2 was inhibited after the cell was transferred with TGF-β1 siRNA. Meanwhile, FGF-2 increased α-SMA expression and improved proliferation, which was associated with p27^kip1^ and cyclin E variation.

**Conclusions:**

The above results suggest that p38/Egr-1 pathway-mediated FGF-2 is involved in 15-HETE-induced differentiation of PAFs into myofibroblasts and cell proliferation.

## Background

Pulmonary vasculature remodeling is the main characteristic of pulmonary artery hypertension (PAH), secondary to chronic obstructive pulmonary disease (COPD) or interstitial lung diseases. Vessel remodeling includes the alterations in the intima, media, and adventitia of the pulmonary artery [1, 2]. Hypoxia is the pathological basis of pulmonary vascular remodeling, especially in the adventitia. It is recognized as the earliest and most prominent structural change during the PAH process [3]. Previous reports indicate that 15-hydroxyeicosatetraenoic acid (15-HETE), one of the most important metabolic products of arachidonic acid (AA) catalyzed by 15-lipoxygenase, is a novel molecule involved in many cellular processes, including cell proliferation, fibrosis, migration, and inflammation [4]. In response to hypoxia, adventitial fibroblast (PAF) is activated and undergoes phenotypic changes, which is followed with proliferation, differentiation, upregulation of contractile and extracellular matrix proteins, and a release of factors that directly affect medial smooth muscle cell tone and growth. PAF transformation is thought to be the key step of hypoxia-induced pulmonary vascular remodeling [5]. 15-HETE induced vascular PAF migration through the activation of STAT3 and stimulated vascular adventitial fibrosis via p38 MAPK-dependent TGF-β1 pathway were clarified by our previous studies [6, 7]. We also identified 15-HETE-induced differentiation of PAF into myofibroblast; however, how and why this happened remains unclear. Thus, we aim to explore the mechanism for 15-HETE-induced PAF phenotypic alteration in this study.

In hypoxia-induced pulmonary hypertension, the early and dramatic increase in the appearance of α-SMA in the adventitia is observed [8]. α-SMA is the most frequently used marker for myofibroblast identification, and the expression of α-SMA in the adventitial vessel wall is a significant signal of fibroblast differentiations into myofibroblast [9, 10]. Myofibroblasts accumulation contributes to the changes in vascular tone and structure under pathophysiological situations. Myofibroblasts are also the most important producers of collagen, fibronectin, tenascin, and elastin in the fibrotic tissues in response to change in the local environment. Thus, myofibroblasts are implicated as key participants in pulmonary vascular remodeling [11].

The stiffening of the pulmonary adventitial layer is recognized as the most sensing response when pulmonary arteries undergo hypoxia. Activation of some growth factors, with subsequent stimulation of protein kinase C and mitogen activated protein (MAP) kinase family members as well as phosphatidylinositol 3-kinase (PI3K), are important regulators of hypoxia-induced PAF proliferation [12]. Some potent mediators in response to earlier hypoxia have been established by a variety of hypotheses. FGF-2 is one of the most interesting growth factors among those regulators such as ET-1, TGF-β, and PDGF. FGF-2 belongs to the fibroblast growth factor family. It is a key player in the process of cell proliferation and differentiation under various stimuli. FGF-2 mediated cell phenotypic changes and increased nuclear p27^kip1^ and FoxO3a through PI3K-Akt and ERK pathways during hypoxia-induced vascular remodeling [13].

Excessive proliferation and migration of PAFs greatly contributed to the pathobiology of PAH [14]. The myofibroblasts could migrate from the adventitia to the media or even the intima, leading to vessel thickening. Myofibroblast accumulation in the intima of arteries has been well documented and is consistently observed accompanying hypoxia in PAH patients [15]. However, where these proliferative cells originate from and who mediates the process is unclear. Here we intend to study the role of 15-HETE in PAF proliferation and cell phenotypic changes, further exploring the underlying mechanisms of 15-HETE in this process.

## Methods

### Reagents

15-HETE was purchased from Cayman Chemicals (Ann Arbor, MI), Anti-phospho-p38, anti-p38, cyclin E, FGF-2, α-SMA, TGF-β1 and GAPDH were purchased from Santa Cruz Biotechnology (Santa Cruz, CA). FGF-2 and Egr-1 siRNAs, FGF-2 and SP600125 were obtained from Sigma, USA. siRNA oligo was synthesized by GenePharma Co., Ltd (Shanghai, China).

### Cell culture

Adventitia from the main pulmonary artery was harvested from fresh rat lung. The pulmonary artery tissue was carefully dissected free of blood vessels and fat under a dissecting microscope, as previous described [6, 7]. Muscular tissue and endothelial cell layers were removed by gentle abrasion of the vessel. The remaining tissue (adventitia) was then dissected into 1 mm^2^ portions. The portions of tissue were evenly distributed over the base of a 25 cm^2^ culture flasks containing 2 ml of Dulbecco’s modified eagle medium (DMEM) with 20 % fetal bovine serum (FBS). The explants were incubated in a humidified atmosphere with 5 % CO_2_ at 37 °C. Fibroblast cells grew from the plants in about 5 days. They were passaged and cultured in 15 % FBS. The 2 to 5 passages were used for the following experiments.

### MTT

PAFs were cultured in a 96-well culture plate (about 1 × 10^4^), and then the cells were transferred with siR15-LO, siRp27^kipl^ or siRNC in DMEM with 5 % FBS. Then the samples were exposed to hypoxia (3 % O_2_). Cinnamyl-3,4-dihydroxy-a’-cyanocinnamate (CDC) or SP600125 at the indicated concentrations were added under hypoxic condition. At the end of the incubation in 37 °C, the cells were incubated for 4 h in a medium containing 0.5 % 3-[4,5-dimethylthiazol-2-yl]-2,5-diphenyl-tetrazolium bromide (MTT), the yellow mitochondrial dye. The amount of blue formazan dye formed from MTT is proportional to the number of surviving cells. The reaction was terminated by adding DMSO to the medium followed by incubation for 10 min. The spectrophotometer absorbance at 540 nm was measured.

### Cell transient transfection

To instigate the knockdown of p27^kip1^, the PAFs were transfected with small interfering RNA, which was designed and synthesized by GenePharma using X-treme Gene siRNA Transfection Reagent (Roche Applied Science, Mannheim, Germany). FAM-tagged non-targeted control siRNA (siRNC) was used to examine and optimize the efficiency of transfection and acted as a negative control. The sense sequences of siRNA and α FAM-tagged non-targeted control sequence were listed below:

TGF-β1: (NM:012775) 5′-CUGACAGCUUUGCGAAUUATT-3′

p27^kipl^: (NM_199501.1) 5′-CCAGGACCUCAAGAAGUUUTT-3′,

NC control: 5′-UUCUCCGAACGUGUCACGUTT-3′.

PAFs were starved with DMEM without serum for 24 h before treatment. 10 μl X-tremeGene siRNA Transfection Reagent were diluted in 90 μl serum-free Opti-MEM-1 medium for 5 min, and 7.5 μl siRNA was diluted with 92.5 μl serum-free Opti-MEM-1 medium, respectively. Then the two above mixtures were mixed for another 20 min at RT. Changed the cell medium for DMEM and added the siRNA and Transfection Reagent mixtures to the cells for 6 h. Cells incubated with siRNC in the same process served as controls. The transfected medium was discarded and cells were cultured in DMEM containing 5 % FBS under normal or 15-HETE stimulation for 24 h. Protein contents were checked with Western blot.

### Immunofluorescence study and microscopic observation

PAFs were cultured on a poly-L-lysine-coated cover glass (15 mm diameter) and washed three times with PBS, followed by fixation with 4 % paraformaldehyde at room temperature (RT) for 15 min. After permeabilization with 0.1 % Triton X-100 for 15 min, the cells were blocked with 3 % normal goat serum at RT for 30 min, followed by incubation with anti-α-SMA primary antibodies (1:100) in PBS at 4 °C overnight. After being washed three times with PBS, the cells were incubated with FITC-conjugated secondary antibody (1:100) diluted by PBS at RT for 2 h and DAPI away from light. Then the cover glass was mounted and examined with the Live cell station (DMI6000B, Leica, Germany).

### Western blot

For Western blot analysis, total proteins were extracted with 400 μl lysis buffer (Tris 50 mM, pH 7.4, NaCl 150 mM, Triton X-100 1 %, EDTA 1 mM, and PMSF 2 mM) and cleaved for 30 min on ice. Then the lysates were centrifuged at 13,500 rpm for 10 min at 4 °C, and the supernatant was collected. The protein concentration in the supernatant was determined by the bicinchoninic acid protein assay (Pierce, Rockford, IL) with bovine serum albumin (BSA) as a standard. 50 μg proteins of each sample were fractionated on a 10 % SDS-polyacrylamide gel. After being electrophoretically transferred to a Pure Nitrocellulose Blotting membrane (Pall Life Science), the membranes were incubated in a blocking buffer (Tris 20 mM, pH 7.6, NaCl 150 mM, and Tween 20 0.1 %) containing 5 % nonfat dry milk powder, and then the blots were probed with primary antibodies overnight at 4 °C, with GAPDH as an internal control. The immunoreactivity was detected using an enhanced chemiluminescence (ECL) and then exposed to X-ray film in a darkroom. Immunoblots were scanned using a V330 densitometer and protein bands were quantified with Quantity One software (EPSON, Bio-Rad Laboratories, Hercules, CA).

### Statistical analysis

All data is presented as mean ± SEM. There are more than two groups of continuous variables in this study, so an one-way analysis of variance ANOVA followed by Bonferroni or Dunnett’s post-hoc test was used for multiple comparisons. A two-tailed value of *p* < 0.05 is considered a statistically significant difference. Data is analyzed using the GraphPad Prism 5.0 and SPSS 14.0.

## Results

### 15-HETE induces PAF phenotypic changes

**Fig. 1:**
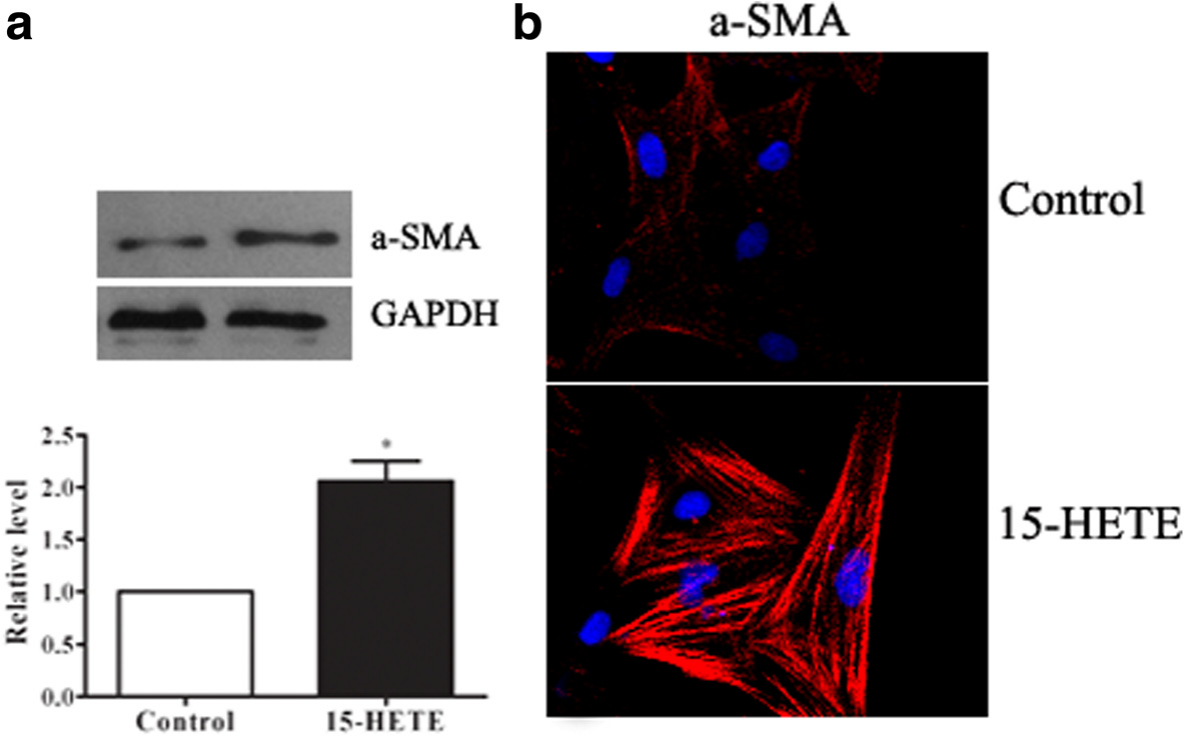
15-HETE induced PAF phenotypic changes. PAFs were incubated with 15-HETE (0.5 μM) for 24 h. Subsequently myofibroblast markers, α-SMA protein expression, was determined as a percentage of the untreated controls using Western blot (**a**) and (**b**). The characteristics of myofibroblasts was assayed by immunocytochemistry staining; the nuclei was stained with 4′,6-diamidino-2-phenylindole (DAPI). Magnification: 200X. Data consists of the means of three independent experiments ± SEM (*n* = 3). * *p* < 0.05 vs. controls

Adventitial fibroblasts differentiate into myofibroblast phenotype under 15-HETE stimulation as our previous studies reported. α-SMA, the most frequently used marker for myofibroblast identification was studied. PAFs were treated with 15-HETE (0.5 μM) for 24 h. As shown in Fig. 1a, cells in the presence of 15-HETE, α-SMA protein levels were significantly increased. Meanwhile, immunofluorescent staining also showed 15-HETE increased α-SMA levels in PAFs (Fig. 1b).

### FGF-2 was involved in 15-HETE-induced cell phenotypic alterations

**Fig. 2:**
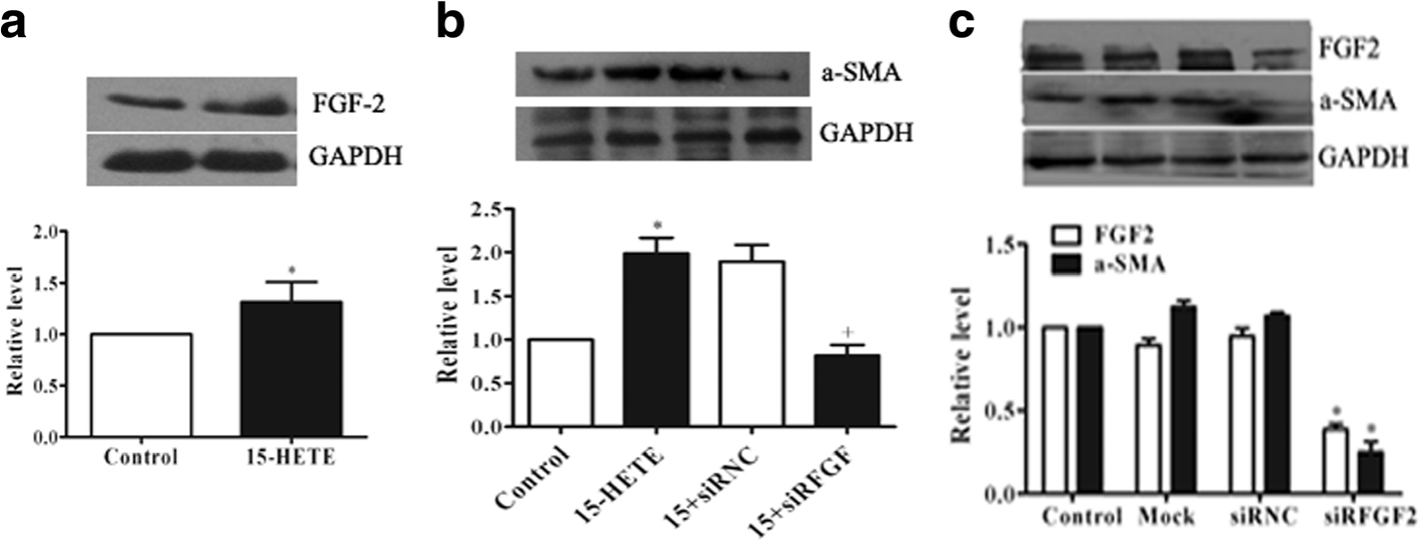
FGF-2 was involved in 15-HETE-induced cell phenotypic alterations. Cells were treated with 15-HETE (0.5 μM) for 24 h. **a** Western blot for FGF-2 expression. **b** Cells were transferred with FGF-2 siRNAs, α-SMA was analyzed by Western blot. **c** The efficiency of FGF-2 silence and its effects on α-SMA without 15-HETE treatments were validated. Data consists of the means of three independent experiments ± SEM (*n* = 3). * *p* < 0.05 vs. controls; +*p* <0 .05 vs. 15-HETE

The differentiation of fibroblasts into myofibroblasts is regulated by a complex microenvironment consisting of growth factors, cytokines, adhesion molecules, and ECM molecules. To examine whether FGF-2 is involved in 15-HETE-induced PAF phenotypic changes, we determined the FGF-2 expression after 15-HETE exposure through immunoblotting. The results showed 15-HETE exposure significantly increased FGF-2 levels (Fig. 2a). Furthermore, silencing FGF-2 with RNAi technique, α-SMA expression was low in cells transfected with siRFGF (Fig. 2b). The efficiency of FGF-2 silence was validated, and silenced FGF-2 also decreased the α-SMA expression without 15-HETE exposure (Fig. 2c). These results suggest that 15-HETE-induced phenotypic conversion of fibroblasts into myofibroblasts was mediated by FGF-2 through detecting α-SMA expression levels.

### 15-HETE-induced FGF-2 upregulation requires p38 MAPK

**Fig. 3:**
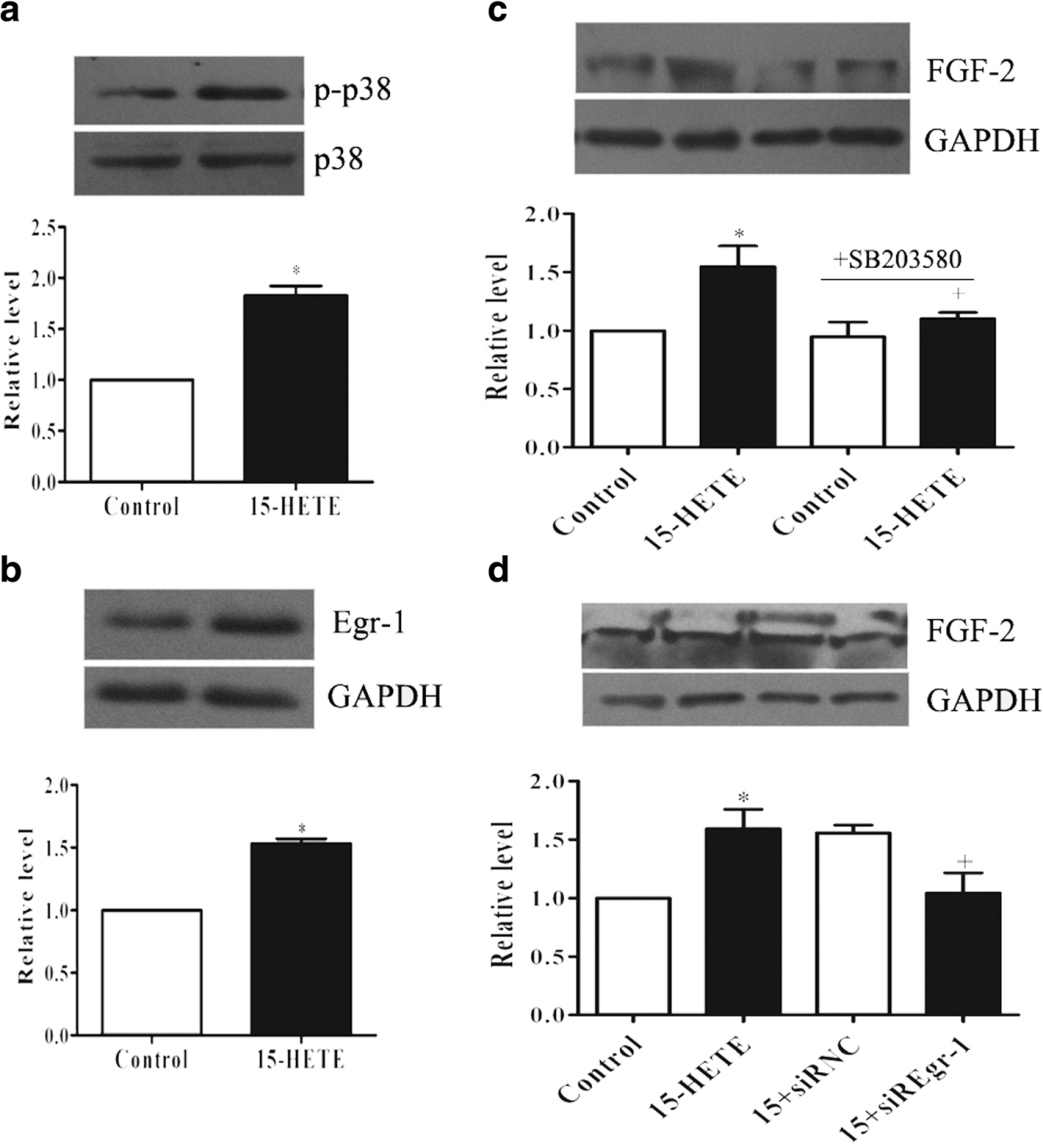
15-HETE-induced FGF-2 upregulation requires p38 MAPK. Cells were treated with 15-HETE (0.5 μM) for 24 h. **a** Western blot for p38 phosphylation and total p38 levels, and p-Egr-1 expression (**b**). **c** Cells treated with p38 specific inhibitor, SB203580 (10 μM), or transfection with Egr-1 siRNA (**d**), FGF-2 was analyzed using Western blot. Data consists of the means of three independent experiments ± SEM (*n* = 3). * *p* < 0.05 vs. controls; +*p* < 0.05 vs. 15-HETE

PAFs were treated with 15-HETE, p38 MAPK and its downstream target Egr-1 activity was analyzed. As shown in Fig. 3a & b, both p38 and Egr-1 phosphorylation levels were raised by 15-HETE stimulation. To determine the effect of p38 on 15-HETE increased FGF-2 expression in PAFs, a pharmacological inhibitor was used. SB203580, an inhibitor of p38 kinase, blocked 15-HETE induced FGF-2 upregulation (Fig. 3c). Egr-1 was able to improve the promoter activity of FGF-2 as reported [16]. So we performed a knockdown of Egr-1, then cells were treated with 15-HETE, and we found that FGF-2 expression was resumed to the control levels (Fig. 3d).

### FGF-2-induced α-SMA expression is mediated by TGF-β1

**Fig. 4:**
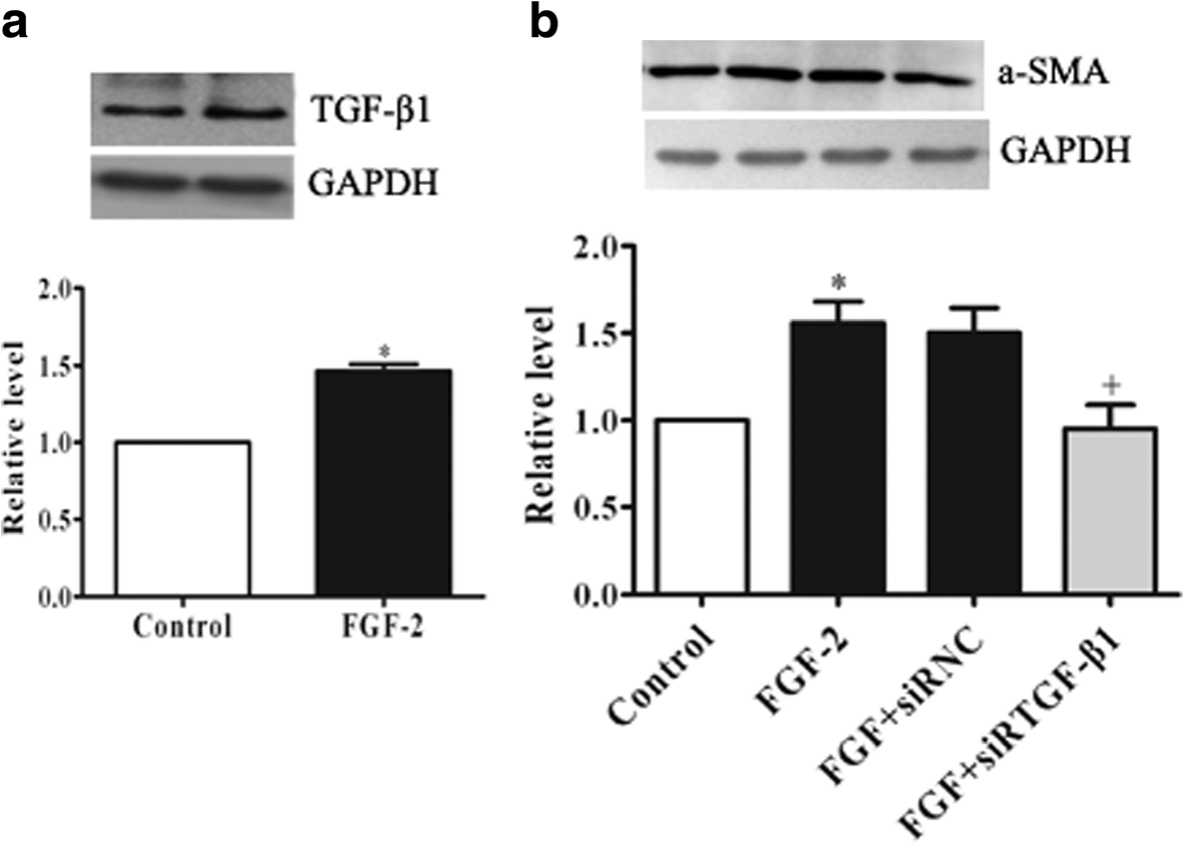
FGF-2-induced α-SMA expression is mediated by TGF-β1. Cells were treated with FGF-2 (15 ng/ml) for 4 h. **a** Western blot for TGF-β1 expression. **b** Cells transferred with TGF-β1 siRNAs, α-SMA changes were analyzed. Data consist of the means of three independent experiments ± SEM (*n* = 3). * *p* < 0.05 vs. controls; +*p* < 0.05 vs. 15-HETE

TGF-β1 is a well-known cytokine capable of inducing the transition of a fibroblast into a myofibroblast phenotype by stimulating α-SMA expression and collagen production [28]. We performed immunoblotting directly for TGF-β1 expression after the cells were treated with FGF-2. Figure 4a showed that FGF-2 exposure (4 h) significantly upregulates the level of TGF-β1 protein in PAFs. To evaluate whether the increase in α-SMA expression induced by FGF-2 is dependent on TGF-β1, PAFs were transiently transfected with TGF-β1 siRNA, and then stimulated with FGF-2. We found TGF-β1 knockdown inhibited FGF-2-induced α-SMA increments in PAFs (Fig. 4b).

### FGF-2 induces α-SMA expression and cell proliferation

**Fig. 5:**
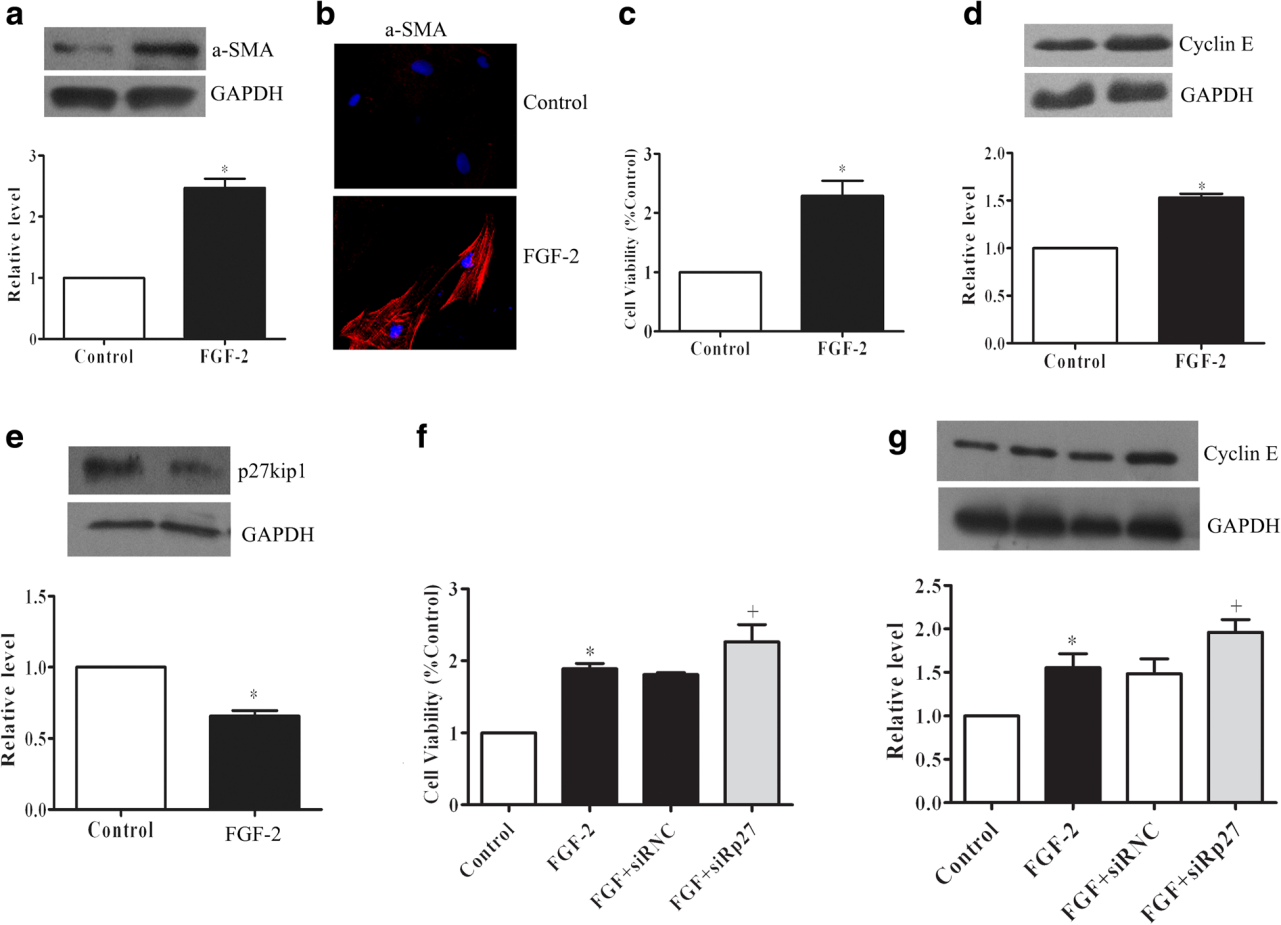
FGF-2 induces α-SMA expression and cell proliferation. Cells were treated with FGF-2 (15 ng/ml) for 4 h. α-SMA protein expressions were determined by Western Blot (**a**) and Immunocytochemistry Staining. The nuclei were stained with DAPI. Magnification: 200X (**b**). **c** Cell viability was measured with MTT, and cell cyclin protein cyclin E (**d**) and p27^kip1^ (**e**) were assayed by Western blot. Cells were transferred with p27^kip1^ siRNAs, cell viability was measured with MTT (**f**), and cylin E was analyzed with Western blot (**g**). Data consists of the means of three independent experiments ± SEM (*n* = 3). *p < 0.05 vs. controls; +*p* < 0.05 vs. 15-HETE

FGF-2-induced α-SMA expressions were assessed by immunoblotting and immunofluorescent staining, the results revealing that FGF-2 upregulated α-SMA levels (Fig. 5a & b). As the transformation of fibroblasts into myofibroblasts always occurs concomitantly with proliferation, we detected the cell viability and cyclin E changes after FGF-2 stimulation. We found that FGF-2 exposure significantly increased cell viability and cyclin E expression in PAFs (Fig. 5c & d).

p27^kip1^ is known inhibitor for cell cycle G1/S transition, and is related to cell proliferation. Therefore, we measured p27^kip1^ levels in FGF-2 treated cells in order to infer the role it plays in cell viability and cyclin E expression. Western blot analysis revealed that, compared with the controls, there is a significant reduction in p27^kip1^ levels after the PAFs’ exposure to FGF-2 stimulation (Fig. 5e). Moreover, transfection with p27^kip1^ siRNA, cell viability and cyclin E were further enhanced in PAFs (Fig. 5f & g). This data suggests that FGF-2 initiates both growth and differentiation signals simultaneously in PAFs.

## Discussion

15-HETE is a metabolic product of AA catalyzed by 15-LO, and it plays a noteworthy role in the pathogenesis of vessel wall diseases [17]. 15-HETE was reported to stimulate VSMC and PAEC proliferation and apoptosis, thus contributing to hypoxia induced pulmonary artery remodeling. Our previous studies also indicated that 15-HETE induced PAF migration and mediated the adventitial fibrosis in the early stage of PAH *in vivo* [4, 6, 7]. Recently, more and more evidence shows that fibroblasts could differentiate into myofibroblasts under hypoxia, which could be critical to hypoxia-induced pulmonary vascular remodeling [18]. However, whether 15-HETE was involved in this pathological process is not clear. In the present study, we investigated the effect of 15-HETE on the PAF phenotypic alteration and explored the underlying mechanisms. We found that 15-HETE upregulated the myofibroblast marker α-SMA, and the FGF-2 expression. The blockage of FGF-2 by siRNA or the inhibition of p38 MAPK relieved the upregulation of α-SMA. Meanwhile, TGF-β1 was required for 15-HETE-induced α-SMA accumulation. Our study indicates that 15-HETE was involved in the cell phenotypic alteration of PAFs, and the TGF-β1 mediated FGF-2 signal pathways could be a novel target for pulmonary vascular adventitial remodeling.

Arterial walls are heterogeneous three-layered structures composed of the intima, media and adventitia [19]. Most studies focus on the endothelial cells and smooth muscle cells (SMC), the principal cellular constituents of the intima and media. Recently, the concept that the vascular adventitia acts as a biological processing center for the regulation of vessel wall function is rapidly emerging, and much more attention is paid to the adventitia and PAFs [20]. In the early stage of PAH, the adventitia is much more sensitive to the environmental stresses such as hypoxia, rapidly expressing and recruiting proteins/factors which perpetuate the inflammatory response. They increase the expression of contractile and extracellular matrix (ECM) protein, differentiating into SM-like cells, which can migrate to the inner layer with greater propensity of proliferation. Meanwhile, the phenotypic alteration and proliferative PAFs synthesize and release inflammatory chemokines and neovascular growth factors, further promoting the vascular angiogenesis and remodeling [21]. In brief, the contribution of adventitial fibroblast phenotype changes to the PAH vascular remodeling cannot be overlooked.

TGF-β1 and FGF-2 are responsible for the phenotypic changes in the activated PAFs. They are the well-known cytokines capable of inducing fibroblast transition into the myofibroblast phenotype by stimulating α-SMA expression and collagen production [22]. It is believed that in hypoxia induced pulmonary vascular remodeling, TGF-β1 and FGF-2 mediate the differentiation of fibroblasts from proliferative phenotype into collagen-production and then into myofibroblastic phenotypes [23]. TGF-β1 induced the isoform switching of fibroblast growth factor (FGF) receptors, causing the cells to become sensitive to FGF-2. FGF-2 is a type of heparin-binding growth factor affecting the proliferation of cells, driving the differentiation of PAFs into myofibroblast through the MAPK and extracellular ERK1 and ERK2 pathways [24]. In the present study, 15-HETE increased the expression of FGF-2 and α-SMA expression. Meanwhile FGF-2 induced upregulation of α-SMA can be blocked by the inhibition of TGF-β1. This data correlated well with our previous studies, indicating that 15-HETE promoted the proliferation of PAFs by the activation of TGF-β1.

The proliferation of PAFs originating in “smooth muscle-like cells” plays a key role in the early stage of cell migration and vessel remodeling. The role of 15-HETE in proliferation of myofibroblast remains unclear. p27^kip1^ is a kind of CDK inhibitor different from the p53 or p16. p27^kip1^ negatively regulates the cell cycle through the inhibition of cyclin enzyme activity, preventing the cells’ G1/S phase transition, and contributes to depressing the cell proliferation [25]. Cyclin E, a regulation actor of CDK2 subunits, mediates the cell cycle G1/S transition. We found that 15-HETE induced the abnormal expression of p27^kip1^, which may lead to G1/S conversion disorder [26]. However, no reports showed the relationship between p27^kip1^ and cell phenotypic alterations in PAFs. This is the first observation in our present study that 15-HETE increased cyclin E expression through inhibiting p27^kip1^ in PAFs. This indicates that 15-HETE induced PAF differentiation via the FGF-2/ TGF-β1 pathway.

## Conclusions

In conclusion, our study suggests that 15-HETE converted adventitial fibroblasts into myofibroblasts through the TGF-β1-mediated FGF-2 signal pathway and stimulated “smooth muscle-like” PAF proliferation based on upregulation of cyclin E induced by p27^kip1^. Our study showed the importance of 15-HETE in PAF cell phenotypic alteration and proliferation under hypoxic stimulation, and it potentiated the therapy target of adventitial remodeling in PAH.
